# Oral sex, cancer and death: sexually transmitted cancers

**DOI:** 10.1186/1758-3284-4-31

**Published:** 2012-06-06

**Authors:** Tahwinder Upile, Waseem Jerjes, Mohammed Al-Khawalde, Hani Radhi, Holger Sudhoff

**Affiliations:** 1 Department of Head and Neck Surgery, Chase Farm & Barnet NHS Trust, Enfield, UK; 2 Head & Neck Unit, University College London Hospital, London, UK; 3 Department of Surgery, School of Dentistry, Al-Yarmouk University College, Baghdad, Iraq; 4 Oral and Maxillofacial Surgery Unit, AL-Mustansirya University’s, Baghdad, Iraq; 5 UCL Department of Surgery, University College London, London, UK; 6 Leeds Institute of Molecular Medicine, Leeds, UK; 7 Oral and Maxillofacial Surgery Unit, Royal Medical Services, Amman, Jordan; 8 Department of Otolaryngology, Head and Neck Surgery, Academic Teaching Hospital of University of Münster, Bielefeld, Germany

## Abstract

We briefly highlight the growing body of recent evidence linking unprotected oral sex with the development of some types of head and neck cancer in younger patients. These tumours appear to be increasing in incidence although the development of more sensitive methods of HPV detection may be a confounding factor.

## Review

The incidence of head and neck squamous cell carcinoma (HNSCC) has been increasing over the last 30 years. It is the 6^th^ leading cause of cancer mortality in the world. Usually these cancers present with advanced metastatic disease causing high morbidity and mortality [1,2]. The poor prognosis of these tumours has remained at around 30–45% despite apparent advances in therapy (i.e. radiotherapy and chemotherapeutic regimens) and confounding by ‘lead time bias’. The most important risk factors so far identified are tobacco and alcohol. These appear to have a synergistic effect on the mucosal surfaces. Rodriguez et al., found a 20-fold increased risk of oropharyngeal cancer below age 46 for heavy smokers, and a 5-fold increase for heavy drinkers; if there was both heavy drinking and smoking, this combination led to an almost 50-fold increase in risk [3].

Interestingly however, in the US the percentage of smokers reduced from 42.5% (1965) to 20.9% (2005) with an associated fall in the traditional subset of HNSCC, however the rates of oropharyngeal cancer especially of the tongue and tonsil have increased again over the last 30 years and are also reflected in European studies [4-7]. This reduction in smoking exposure was also found in RTOG 9003 and calls into question: how much of the improvements in absolute survival observed in clinical trials over time are due to changes in therapy over time rather than being driven by social factors? [8]. A recent study by Jerjes et al. came to the conclusion that tobacco and alcohol reduction/cessation at time of diagnosis tends to reduce mortality in oral cancer patients [9].

Viral genes interfere with cell replication control mechanism. Many other viruses are known to predispose to tumours (Table 1). Viruses associated with tumors can be classified into two types depending on the nucleic acid in the viral genome and the nature of strategy to induce malignant transformation. The RNA tumor viruses (retroviruses), when they infect cells, the viral RNA is copied into DNA by reverse transcription and the DNA is introduced into the host genome, where it persists and can be inherited by subsequent generation of cells. Transformation of the infected cells can be traced to oncogenes that are carried by the viruses. The viral oncogenes are closely similar to cellular genes, the proto-oncogenes, which code for components of the cellular machinery that regulates cell proliferation, differentiation, and death. While, DNA viruses replicate lytically and kill the infected cells. Transformation happens in non-permissive cells where the infection cannot advance to viral replication. The transforming capability of DNA tumor viruses has been traced to numerous viral proteins that work together to stimulate cell proliferation, overriding some of the normal growth regulator mechanisms in the infected cell and its progeny. Different from retroviral oncogenes, DNA virus oncogenes are indispensable components of the viral genome and have no counterpart in the normal host cells. Some of these viral proteins bind to the protein yields of two key tumor suppressor genes of the host cells, the retinoblastoma gene and the p53 gene, deactivating them and thereby permitting the cell to replicate its DNA and divide [1-9].

**Table 1:** Viruses are known to predispose to tumours (some examples are included in the table)

**Hepatitis B virus and liver adenocarcinoma**
EBV, Burkett’s lymphoma and nasopharyngeal carcinoma
HIV and Non-Hodgkin’s lymphoma, Kaposi sarcoma…etc.
HPV and cervical cancer

Recent data have now attributed a human papillomavirus (HPV) aetiology to a subset of head and neck cancers particularly tongue base (lingual tonsils) and palatine tonsils (oral cavity) [8,10]. HPV has also been implicated in nearly 100% of cervical cancers, 66% of anal cancers, 43% of vaginal/vulval cancers, 44% of penile cancers and almost 15% of oropharyngeal tumours [11] and are implicated in up to 60% of head & neck cancers in the US [8].

The HPV family of DNA-viruses preferentially infects squamous epithelial cells with over a 100 separate genotypes and more than 40 of which cause genital infections. They represent the most common sexually transmitted disease worldwide with nearly 80% of infection rates in sexually active adults in the US of at least one HPV type by the age of 50 years. The peak prevalence occurs during early sexual activity (adolescence and young adulthood) which declines with age with HPV 16 being the most common [12]. Most people who get the infection do not develop sequalae; but a minority develops cancer as a consequence of the infection. HPV can induce a spectrum of epithelial disease ranging from asymptomatic infection, simple papilloma through to frank squamous cell carcinoma (SCC). The virus tends to manifest at epithelial junctional areas (i.e. where the epithelium changes from stratified squamous to simple cuboidal, collumnar or contains lymphoid tissue). This may also coincide with areas of differing embryological origins. Common areas affected are the oropharynyx, including the tonsils and tongue base. These areas are notorious as residences for ‘occult’ primaries, usually presenting at a more advanced stage in younger patients.

Human papillomavirus (HPV) as a risk factor was first suggested in 1983 by Syrjänen et al., who noted that 40% of the studied cancers contained histological and morphological similarities with HPV-associated lesions [13]. Subsequently, there is growing evidence strongly support a role for HPV in a subset of HNSCC. These head and neck cancers tends to occur in younger people, originates from the oropharynx (particularly the tonsils), and are poorly differentiated, and are related to sexual practices.

Work by D’Souza and colleagues recently showed HPV infection is likely to be sexually acquired [14] with increased risk of oropharyngeal cancer with either many (more than 26) lifetime vaginal-sex partners or six or more lifetime oral-sex partners. Obviously the issue is confounded by the use of and availability of mucosal protection for genital rather than oral intercourse (Tables 2 and 3).

**Table 2:** HPV infection is likely to be sexually acquired: vaginal-sex and oral sex partners

**Sexual behavior**	**Control patients**	**Patients with Oropharyngeal Cancer**	**Adjusted Odds ratio (95% CI) HPV 16+ Patients**	**Adjusted Odds ratio (95% CI) All patients**
Lifetime number of vaginal-sex partners				
0–5	54	31	1.0	1.0
6–25	32	41	2.7 (1.4–5.5)	2.2 (1.2–4.0)
**≥26**	**14**	**28**	**4.2 (1.8–9.4)**	**3.1 (1.5–6.5)**
Lifetime number of oral-sex partners				
0	19	12	1.0	1.0
1–5	55	46	3.8 (1.0–14.0)	1.9 (0.8–4.5)
**≥6**	**26**	**42**	**8.6 (2.2–34.0)**	**3.4 (1.3–8.8)**

Modified from *D’Souza G, Kreimer AR, Viscidi R, Pawlita M, Fakhry C, Koch WM, et al. Case-control study of human papillomavirus and oropharyngeal cancer. N Engl J Med. 2007 May 10;356*(19)*:1944–56.*

**Table 3:** Most common types of infection associated with cancer are HPV-16 and to a lesser extent 18

**Measures of HPV**	**Prevalence (%)**		**Odds SimplePara> HPV 16+ Patients**
	**Control**	**Oropharyngeal cancer**	**Adjusted compared to control (1.0)**
HPV-16 serology	7	57	32.2 (14.6–71.3)
Oral HPV-16 infection	4	32	14.6 (6.3–36.6)
All Oral HPV infection	6	37	12.3 (5.4–26.4)
HPV-16 E6 or E7 serology	4	64	58.4 (24.2–138.3)

Modified from *D’Souza G, Kreimer AR, Viscidi R, Pawlita M, Fakhry C, Koch WM, et al. Case-control study of human papillomavirus and oropharyngeal cancer. N Engl J Med. 2007 May 10;356*(19)*:1944–56.*

Establishing the evidence for a causal role for HPV in a subset of HNSCC has been compounded by the heterogeneity of the tumours and multiple primary anatomical sites. These studies employed different detection techniques; different sampling methods and different storage procedures. The non-standardization of these multiple variables has confused the issue [1].

Serological and molecular markers of HPV infection are also associated with increased risks of HNSCC. Hansson et al. found a strong association between the detection of high-risk HPV DNA in the oral cavity and oropharyngeal carcinoma (OR 230; CI 45–1200) after adjusting for alcohol and tobacco usage [15].

The most common types of infection associated with cancer are HPV-16 [16] and to a lesser extent 18 (Table 4) but other high risk mucosal types exist. Mork et al. demonstrated an increased risk of greater than 2-fold for subsequently developing oral cancer if there was HPV-16 seropositivity. The rates of HPV-16 infection have increased from 23% (1970s) to 68% (2000’s), although this may simply reflect more sensitive detection methodologies [17]. It is estimated that individuals with an oral HPV 16 infection have between a 15- and 200-fold increase in risk of developing oropharynx cancer [8]. Oral sex with multiple partners is one of the significant risk factors for oral cancer and oropharyngeal cancer. Young people, who increasingly practice oral sex especially with many partners, may be driving the increase in these cancers.

**Table 4:** HPV infection is likely to be sexually acquired: systematic meta-analysis

**Systematic meta-analysis**	**Oropharyngeal cancer**	**Oral cancer**	**Laryngeal cancer**
HPV prevalence (%)	35.6	23.5	24
HPV 16 prevalence (%)	86.7	68.2	69.2
HPV 18 prevalence (%)	1	8	3.9

Modified from *Kreimer AR, Clifford GM, Boyle P, Franceschi S. Human papillomavirus types in head and neck squamous cell carcinomas worldwide: a systematic review. Cancer Epidemiol Biomarkers Prev. 2005 Feb;14*(2)*:467–75.*

The pathogenesis of viral induced oncogenesis is presumably similar to cervical cancer development but heterogeneity and the existence of multiple pathways to carcinogenesis is highly likely. This is also likely to reflect a difference in life cycles of the different HPV subtypes in different mucosal locations, with an associated difference in mucosal immune responses [1]. It must be remembered that the oral cavity is a battlefield of healing mucosal micro abrasions which could in the right circumstances of altered local host defenses allow viral inculcation, infection and entrenchment leading to somatic genetic change. Changes in immuno-tolerance [16] at these ‘special’ immuno-modulating sites (the oropharyngeal parts of Waldeyers ring of lymphoid/squamous tissue) combined with further environmental triggers then lead to cancerous changes. Basically, viral “genes load the gun and environment pulls the trigger”.

The high risk human papilloma viruses (types 16 and 18) contain the transforming genes E6 and E7. Although these gene products have a multitude of effects, in essence E6 binds to and inactivates the p53 suppressor gene resulting in tolerance of DNA errors and tumour entrenchment. Whilst E7 binds the RB suppressor gene, causing a loss of control over the cell cycle resulting in continuous proliferation. This interaction between viral derived products and intrinsic cellular proteins inhibits apoptosis in infected cells, enabling them to both survive and undergo continuous growth which inhibits differentiation. This has been found quite readily in oral keratinocytes and epithelial cells [18,19].

## Clinical implications of HPV + in head and neck cancers

Accumulating evidence suggests that HPV + status is an important prognostic factor associated with a favourable outcome after treatment in head and neck cancers [19-23]. Prospective multi-centre clinical study (as opposed to the retrospective studies previously discussed), Gillison et al. demonstrated that patients with HPV + tumours had better response rates after induction chemotherapy (82% vs. 55%), and after chemoradiation treatment (84% vs. 57%) compared to patients with HPV- tumours. Patients with HPV + tumours had an improved overall survival of 33%, and after adjustment for age, tumour stage and ECOG status, a lower risk of progression and death from any cause, compared to those with HPV- tumours [24]. About 85% of patients with HPV+ tumors are still alive within 5 years of their cancer diagnosis, compared with about 45% of those with non-HPV tumors [8]. These studies provided strong evidence that HPV + tumour status was both associated with a better response to current treatment regimes, but also with a much improved survival rate, and risk of progression, compared to HPV- tumour status. It maybe that the HPV + tumours are different in both their aetiology and biology from the purely smoking and alcohol associated HPV- tumours, hence their differing response to treatment [8,25]. The better prognosis and treatment responses to chemotherapy and radiotherapy by HPV + tumours may mean that HPV status detection is required to better plan and individualise patient treatment regimes. It is now common practice to send initial tumour biopsy material for HPV sub-typing and fast track these patients through chemoradiation schedules with anti-angiogenic antibody.

Finally, it may be that the subgroup of head & neck tumours often in younger patients are induced by sexually transmitted viruses and we would advice caution to oral-sex practitioners and promote the use of barrier methods (condoms, oral dams). We would also advocate the early specialist referral of any patient presenting with i.e. persistent unilateral oropharyngeal symptoms or signs (>3weeks) even in the absence of the ‘traditional predisposing factors’ (smoking, alcohol, age) to head & neck squamous cell carcinoma. One would welcome vaccination schedules which include the HPV subtypes associated with head and neck cancer. Unfortunately not all the commercially available vaccines include the subtypes in question although there is some evidence of limited cross-over protection.

## Competing interests

The authors declare that they have no competing interests.

## Authors’ contributions

TU, WJ, MA and HR designed the study, carried out the literature research and manuscript preparation. TU, WJ, MA, HR and HS were responsible for critical revision of scientific content and manuscript preparation and review. All authors contributed to conception and design and approved the final version of the manuscript.
